# Posterior Segment Optical Coherence Tomography in Uncooperative Paediatric Patients Using Exo-Illumination and Microscope-Integrated Optical Coherence Tomography

**DOI:** 10.7759/cureus.32994

**Published:** 2022-12-27

**Authors:** Ankur Singh, Mohit Dogra, Bruttendu Moharana, Ramandeep Singh

**Affiliations:** 1 Department of Ophthalmology, University College of Medical Sciences, Delhi, IND; 2 Advanced Eye Centre, Postgraduate Institute of Medical Education and Research, Chandigarh, IND; 3 Department of Ophthalmology, All India Institute of Medical Sciences, Bhubaneswar, IND

**Keywords:** optical coherence tomography (oct), microscope-integrated oct, paediatric retina, examination under anaesthesia, surgical retina

## Abstract

Background and Objective: To describe a non-invasive technique for the acquisition of retinal optical coherence tomography (OCT) scans in paediatric patients undergoing examination under general anaesthesia (EUA) using microscope-integrated OCT (MIOCT).

Study Design: Prospective observational study

Methods and Material: The study included 10 paediatric patients undergoing EUA for posterior segment pathology. These patients underwent OCT using MIOCT. No sclerotomy was made during imaging. The fundus was externally illuminated with a 25 gauge endoilluminator probe placed at the limbus and directed towards the posterior pole to aid in image acquisition by MIOCT (exo-illumination). Imaging for all patients was done by two trained vitreoretinal surgeons independently. Acquisition time was recorded for each surgeon. Interobserver variability in acquisition time and image quality was assessed to estimate the reliability of the novel imaging technique.

Results: In nine cases (90%), MIOCT successfully imaged the posterior segment pathology while in one case (10%) of X-linked retinoschisis, it failed to detect an inner retinal break located anteriorly at the equator. The mean acquisition time for surgeons one and two was 211.75 ± 26.00 and 212.58 ± 23.47 seconds, respectively. There was no significant difference in total image acquisition time between the two surgeons (P = 1.0) and the findings of both surgeons were comparable for structural morphology. 4x4 mm-sized scans provided the best delineation in macular pathology, while a 16x16 mm scan size was best suited for localising the area of interest and post-equatorial pathology.

Conclusion: Using this technique acquisition of posterior segment OCT scans can be achieved non-invasively, using exo-illumination and MIOCT in paediatric patients undergoing EUA.

## Introduction

In current ophthalmic practice, optical coherence tomography (OCT) is the preferred imaging modality for retina specialists [1]. It has the ability to provide in-vivo, quasi-histological images of the ocular tissues with brilliant spatial resolution [2]. Since its inception in ophthalmology, it has proven to be a vital diagnostic and therapeutic surveillance tool for a wide spectrum of the retina and choroidal as well as anterior segment pathologies in adults. Until recently, infants and young children were deprived of this technology, as conventional table-mounted OCT devices are not designed for infants and young children. Portable handheld OCT has overcome these limitations as they are optimised for use in the paediatric population [3]. Images obtained by handheld OCT (HHOCT) have not only added to our understanding of the normal development of the fovea and the optic nerve head in infants, but also demonstrated previously unrecognised structural features including cystoid macular oedema, epiretinal membranes, blood vessel anomalies, punctate hyperreflective vitreous opacities, vitreous bands, and retinoschisis in infants with retinopathy of prematurity [4,5]. HHOCT has also been used to study optic nerve hypoplasia, retinal detachment, retinoschisis, retinoblastoma, optic pathway gliomas and many other ocular conditions [6]. However, the portable OCT system does pose unique challenges during image acquisition such as the need for manual alignment, inter-examiner variability and image losses due to probe tilt, hand movement and infant eye movement, this affects the overall reliability of this system [7]. In addition to this, the added cost of the machine and a protracted learning curve for acquiring images add to the list of challenges associated with this system [8].

HHOCT can also be used intraoperatively, however, intraoperative use of HHOCT has been shown to significantly increase the surgical time, as surgeons need to shift between microscope and OCT, to scan the area under interest during a surgical procedure [9]. Other challenges with the HHOCT system include motion artefacts, difficult stabilization, and lack of real-time imaging. This led to the development of microscope-mounted OCT initially, and later integration of the OCT system into the microscope itself. Microscope-integrated OCT (MIOCT) system allows for real-time visualization of instrument-tissue interactions during vitreoretinal surgery. This allows the surgeon to perform image-assisted and image-guided surgical manoeuvres thereby enhancing surgical outcomes [10,11]. Multiple studies have examined the feasibility, utility, and safety of MIOCT across multiple ophthalmic surgeries and have found that MIOCT, aided in identifying subclinical pathology intraoperatively, affecting surgical management and thereby improving surgical outcomes [10-13]. All these benefits have made MIOCT an essential tool for vitreo-retina surgical management. However, a prerequisite for MIOCT image accusation is the need for an illumination source in the vitreous cavity for visualization of the area of interest. This acts as a limitation for MIOCT in comparison to HHOCT and limits the use of MIOCT in paediatric patients undergoing examination under general anaesthesia (EUA) as MIOCT requires the creation of a sclerotomy port for an intraocular light source.

The index study aims to describe a novel non-invasive MIOCT image acquisition technique for posterior segment pathology in paediatric cases during EUA. This obliviates the need to create a sclerotomy and provides real-time OCT images of the retina in the absence of HHOCT.

## Materials and methods

This was a prospective observational consecutive case series. Consecutive paediatrics patients being referred between January 2019 and March 2019 to Advanced Eye Center, Postgraduate Institute of Medical Education and Research with suspected posterior segment pathology and uncooperative for retinochoroidal imaging on conventional table-mounted OCT were enrolled in this study. Institutional Ethics Committee, Post Graduate Institute of Medical Education & Research, Chandigarh’s approval was obtained before the conduct of the study (INT/IEC/2018/036). The study adhered to the tenets of the Declaration of Helsinki and the rules laid down by the Health Insurance Portability and Accountability Act of 1996. Written informed consent was obtained from the parents/legal guardians of all participants at the beginning of the study.

Demographic features, past and recent medical records including relevant history associated with referral were documented for all the subjects, included in the study. All recruited patients underwent ophthalmic evaluation during EUA that included intraocular pressure measurement, and anterior segment examination under the operating microscope followed by dilated posterior segment visualization with a binocular indirect ophthalmoscope. The posterior segment findings were recorded and the area to be imaged was identified. Patients having media opacity, that might preclude retinochoroidal visualization and imaging were excluded from the study. Patients whose parents/legal guardians did not consent to the procedure were also excluded from the study. Vitreous and retinochoroidal microstructural imaging was done with MIOCT using the exo-illumination technique, by two trained vitreoretinal surgeons (AS, BM) independently, for all enrolled patients. The total image acquisition time (TIAT) for both surgeons was recorded.

Imaging technique

Patients under general anaesthesia were imaged using MIOCT (RESCAN 700, Zeiss, Oberkochen, Germany) in a supine position on the operation theatre table. EUA was performed and the area of interest was identified by dilated indirect ophthalmoscopic examination. The operating microscope (OPMI LUMERA 700 with RESCAN 700, Zeiss, Oberkochen, Germany) with the wide-angled viewing system (RESIGHT 700, Zeiss, Oberkochen, Germany) was then used along with a 25-gauge (G) endoilluminator probe. The probe was held perpendicular to the limbus, at the 3 o’clock meridian for the left eye and 9 o’clock meridian for the right eye, with the light beam directed towards the macula to provide external illumination. As soon as the optic disc was visualised in the eyepiece, fine focusing of the operating microscope was utilised to enhance visualization of the disc and retinal vasculature to achieve an appropriate focal plane. At this point, MIOCT was switched on. The cube image acquisition protocol was selected and OCT scans of the retina were obtained and viewed in real-time on ZEISS CALLISTO PC. Scan quality was improved using the inbuilt autofocus tool as well as by manually altering the OCT signal strength. This process was repeated until good-quality scans were achieved with clear demarcation of retinal layers. A drop of artificial tears was added to provide stable tear film if needed for good-quality image acquisition. With the help of inbuilt location arrows, the cross-hair of the cube image acquisition protocol was moved to visualise the fovea. Scan length was altered in steps (4x4, 8x8, and 16x16) based on the diagnosis (range 3 mm-16 mm). The OCT cube was rotated 90 degrees to obtain visualise the area of interest. Once the area of interest was identified, the cube size was decreased to 4x4 mm size and the cross-hair was moved with location arrows to the area of interest. Any manual or auto-focusing if required was done. The OCT cube was again rotated 90 degrees to obtain more detailed scans of the area of interest. Scan length was again altered based on the diagnosis if required. Acquired scans were captured and saved as digital imaging and communications in medicine (DICOM) files, which were reviewed later on. We labelled this as the “exo-illumination MIOCT technique” for obtaining non-invasive retinal OCT images in children under general anaesthesia.

Image acquisition time

TIAT was defined as the total time taken by the surgeons to acquire MIOCT images which included accusation time for all scans of different sizes, cube rotation time and time elapsed during movement of cube cross-hair from the fovea to the area of interest. The TIAT for both surgeons was recorded.

Image evaluation

DICOM files for both surgeons were converted to images and saved. These saved images were reviewed for image quality by two independent observers (MD, RS). Images that allow for the delineation of layered retinal structure and retinochoroidal pathology were recorded as “good quality images” while the rest were recorded as “inferior quality images”. The images excluding the inferior quality images were evaluated by the two observers for any vitreous, retinal and choroidal structural abnormality in all 4x4 mm, 8x8 mm, and 16x16 mm scan sizes. All scans of different sizes, centred over the area of interest for each patient were compared with each other. The scan size or sizes providing the most useful information about the pathology was selected. Discrepancies between the observers were resolved by open adjudication. Findings of the both observers were recorded.

Statistical analysis

Statistical analysis was performed using GraphPad Prism (GraphPad Software Inc, La Jolla, CA). The differences in mean total acquisition time for two surgeons were calculated using an independent t-test. Qualitative descriptions of the imaging characteristics were performed. The level of significance was set at 0.05.

## Results

Ten eyes of 10 patients (seven males and three females) fulfilled the inclusion criteria and were enrolled in the study. The mean age of the subjects was 4.2 (range: 3-5) years. One patient referred for posterior segment evaluation had a history of previous retinal surgery for retinal detachment (RD). Patient diagnosis and demographic profile are summarised in Tables 1-2.

**Table 1:** Diagnosis, MIOCT imaging findings and best scan size for imaging. S. No: serial number; LE: left eye; RE: right eye; MIOCT: microscope-integrated optical coherence tomography; SRF: subretinal fluid *Case 2 in which MIOCT could not image the equatorial inner retinal break.

S. No.	Posterior segment pathology	Laterality	Structural alteration on MIOCT	Scan sizes best suited for imaging of the pathology in (mm)
1	Post-traumatic macular scar	LE	Macular scar with disruption photoreceptor layer and retinal atrophy	4x4
*2	Juvenile retinoschisis	RE	Foveal retinoschisis	4x4 and 16x16
3	Coloboma with retinal detachment	LE	Identification of normal intercalary membrane	4x4 and 8x8
4	Superior retinal detachment	LE	Superior retinal detachment with foveal involvement with attached inferior parafovea	4x4
5	Post-traumatic macular pathology	RE	Foveal and parafoveal retinal atrophy	4x4
6	Retinal detachment	LE	Determination of SRF (preoperative), determination of residual SRF (immediate post-surgery) and attached macula with normal foveal contour (at one-month follow-up)	8x8 and 16x16 (preoperative), 8x8 and 16x16 (immediate post-surgery) and 4x4 and 8x8 (at one-month follow-up)
7	Juvenile retinoschisis with retinal detachment	RE	Retinal detachment and retinoschisis	16x16
8	Disc coloboma with retinal detachment	LE	Nasal retinoschisis with foveal detachment	4x4 and 16x16
9	Traumatic subretinal haemorrhage with foveal sparing	LE	Extent and height of subretinal haemorrhage and relative foveal sparing	4x4 and 8x8
10	Follow-up case of retinal detachment	RE	Attached macula with normal foveal contour	4x4, 8x8, 16x16

**Table 2:** Demographic profile of patients and total image acquisition time for surgeons. S. No: serial number; M: male; F: female *Case 2 in which microscope-integrated optical coherence tomography could not image the equatorial inner retinal break #Case 6 of retinal detachment where exo-illumination microscope-integrated optical coherence tomography technique was used to image the retina, pre-scleral buckling, immediate post-scleral buckling and on 3-month follow-up P-values of p<0.05 were considered statistically significant

S. No.	Sex	Age (years)	Total image acquisition time of surgeon one in (seconds)	Total image acquisition time of surgeon two in (seconds)	P value
1	M	3	218	224	1.000
*2	M	5	268	266	
3	M	4.5	212	204	
4	M	3.5	215	212	
5	M	4	174	183	
#6	F	3	218; 208; 180	217; 212; 184	
7	M	5	228	224	
8	F	4	212	210	
9	M	5	178	184	
10	F	5	230	231	

**Figure 1:**
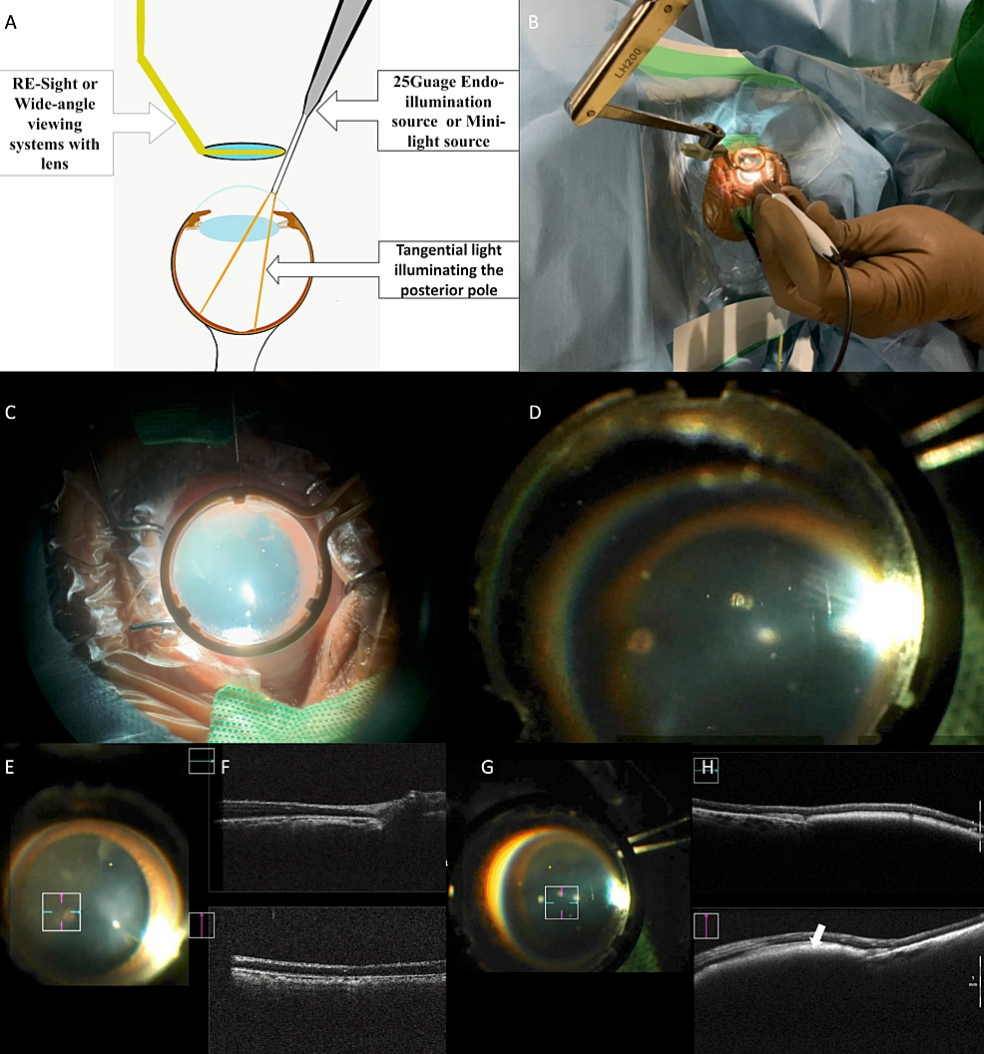
Exo-illumination MIOCT technique with a representative case. A: Diagrammatic representation of the “Exo-illumination technique” showing the placement of RESIGHT 700 viewing system over the eye while the retina is illuminated using a 25G endoilluminator placed at the limbus, with the light beam directed tangentially and posteriorly (authors’ own creation); B: external view and; C: surgeon’s view showing the position of the endoilluminator and the viewing system; D: surgeon’s view during the procedure showing the optic disc and surrounding retina being visualised through the viewing system with the external light source and; E: the cube scan of the MIOCT focused just nasal to the optic disc; F upper image: MIOCT horizontal cross-sectional 4x4 mm size OCT scan showing normal retinal architecture with the optic disc at one end while; F lower image: the vertical OCT shows normal retinal architecture; G: a cube scan of the MIOCT focused just above the fovea; H upper image: MIOCT horizontal cross-sectional 8x8 mm size OCT scan of a patient (Case 9) showing neurosensory retinal architecture with subretinal haemorrhage with subtle neurosensory retinal detachment while the; H lower image: vertical OCT also shows subretinal haemorrhage (arrow). MIOCT: microscope-integrated optical coherence tomography

Both the surgeons using the exo-illumination MIOCT technique could delineate the foveal microstructure for all 10 eyes (100%). In nine eyes (90%) OCT scans obtained by both surgeons showed the area of interest (Figure 1). Foveal and parafoveal imaging using exo-illumination MIOCT technique in three patients with traumatic macular pathologies revealed the extent of atrophic scarring along with disorganised outer retina in two, while subretinal haemorrhage with relative foveal sparing in one. In patients with RDs (n=5), the macular assessment revealed foveal neurosensory detachment along with nasal retinoschisis in a patient with optic disc coloboma and retinal detachment (RD) (Case 8). While in another patient foveal retinoschisis was seen as continuing anteriorly with RD (Case 7). In a case with a previous history of RD surgery, the normal foveal microstructure was seen during imaging which followed amblyopia treatment. While in one case of X-linked retinoschisis (Case 2) with an inner retinal break at the equator, exo-illumination did not work and consequently, MIOCT was not able to scan the area of interest. On evaluation by both observers, none of the scans obtained by the surgeons using the exo-illumination MIOCT technique met the criteria for the inferior quality scan. All scans had good spatial resolution and could delineate the retinal layered structure as well the retinal pathology. Table 1 summarises the clinical diagnosis and imaging characteristics of all the cases.

**Figure 2:**
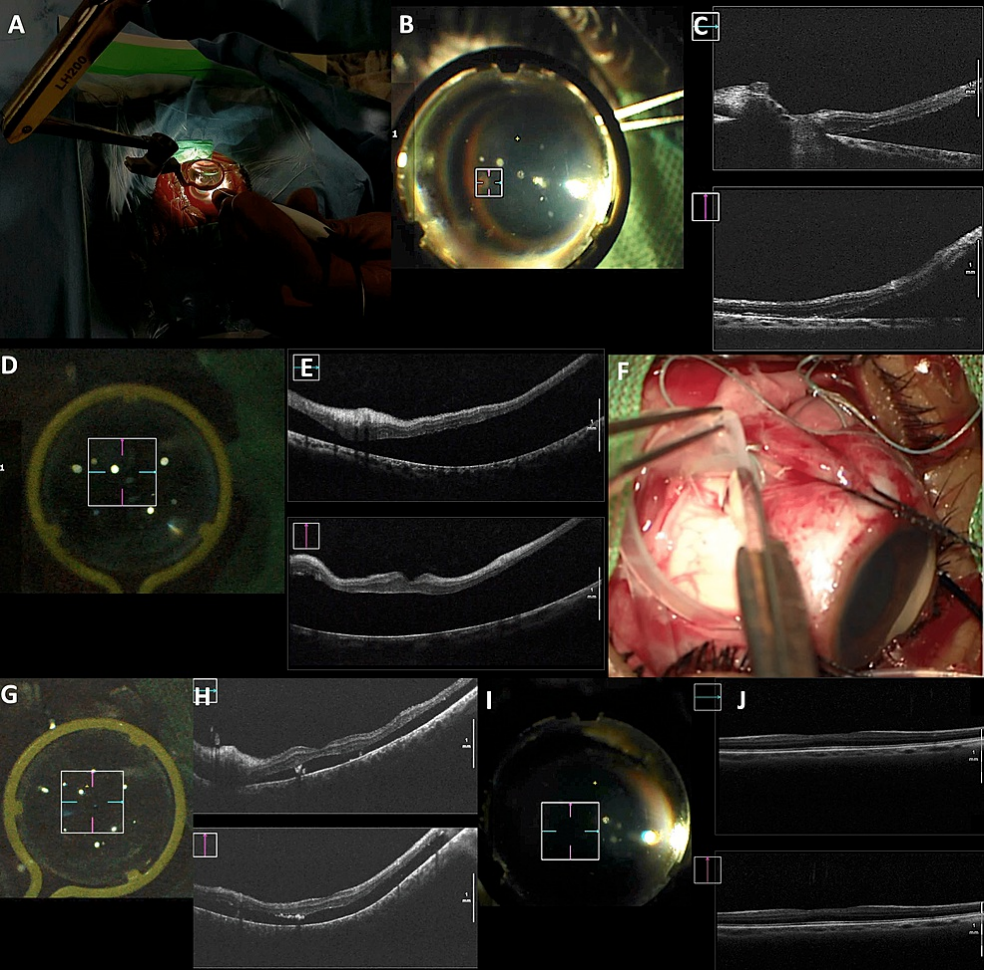
Preoperative, immediate post-surgery and follow-up assessment in a patient with retinal detachment (Case 6) using exo-illumination MIOCT technique. A: External view showing the position of the endoilluminator and the viewing system; B: the surgeon’s view during the procedure showing the optic disc and surrounding retina being visualised through the viewing system with the external light source and a 3x3 mm scan size cube scan of the MIOCT focused just temporal to the optic disc; C upper image: MIOCT horizontal cross-sectional OCT scan showing retinal detachment and optic disc at one end while; F lower image: the vertical OCT shows superior retinal detachment with attached juxtapapillary inferior retina; D: MIOCT cube scan cross-hair of 8x8 mm scan size centred just above fovea; E upper image: the horizontal cross-sectional OCT scan showing retinal detachment while; E lower image: vertical OCT through fovea shows retinal detachment; F: surgeon’s view showing scleral band and creation of sclerotomy for subretinal fluid drainage; G: MIOCT cube scan cross-hair of 8x8 mm scan size centred just on the fovea; H upper image and lower image: the horizontal and vertical cross-sectional OCT scan showing a reduction in neurosensory detachment height at the end of surgery; I: 3 months post-operative, MIOCT cube scan cross-hair of 4x4 mm size, at the fovea; J upper and lower image: showing attached retina with normal foveal architecture in both horizontal and vertical scan. MIOCT: microscope-integrated optical coherence tomography

Optic disc, fovea and parafoveal microstructural changes were best seen with a 4x4 mm scan size. 16x16 mm scan size was used for localising the area of interest as well as giving an overview of the extent of the lesion. The extent of RD, foveal involvement or sparing as well as quantification of subretinal fluid was best done in 16x16 mm scans. As in a patient of retinal detachment (Case 6), pre-scleral buckling 16x16 mm and 8x8 mm OCT images showed foveal neurosensory detachment, immediate post-scleral buckling and subretinal fluid drainage, OCT images showed a decrease in the height neurosensory detachment. On follow-up using an 8x8 mm scan, images of this patient revealed an attached retina with normal ellipsoid zone architecture (Figure 2). Clinical diagnosis with scan sizes for providing the best delineation of pathology are summarised in Table 1.

The mean TIAT for surgeons one and two was 211.75 ± 26.00 and 212.58 ± 23.47 seconds, respectively for all patients. The mean TIAT for both surgeons was comparable with no significant difference. The TIAT for surgeons one and two ranged from 174-268 to 183-266 seconds, respectively and varied with the type of retinal pathologies. The TIAT for surgeons one and two are summarised in Table 2.

## Discussion

This index study describes a non-invasive exo-illumination MIOCT technique, for imaging the retinochoroidal architecture in paediatric patients during EUA without the need for a sclerotomy, thereby maintaining a closed chamber. In our study, both surgeons could successfully image the retina of all patients (100%) using the exo-illumination MIOCT technique, with 90% of scans showing the area of interest. All scans acquired with this technique had good spatial resolution with clear delineation of retinochoroidal microstructure. In a patient (Case 3) with RD and retinochoroidal coloboma, MIOCT visualised a normal intercalary membrane thereby ruling out a break in the intercalary membrane, as the cause of RD. Retinal breaks within the intercalary membrane are seen in 80% of coloboma-associated RD [14]. Due to the lack of contrast, retinal breaks within the intercalary membrane are difficult to visualise by conventional indirect ophthalmoscopy [15]. Therefore, it is prudent to carefully examine intercalary membranes in RDs associated with coloboma. OCT allows for clear delineation of the intercalary membrane and the margin of retinochoroidal coloboma [16].

OCT can also allow for easier differentiation of retinoschisis from RD [17]. In eyes with juvenile retinoschisis with RD, the anterior edge of the schisis-detachment cavity may continue with complete separation of the retina from retinal pigment epithelium as seen in a patient (Case 7) in our study, on MIOCT scan. Foveal assessment is a prerequisite to rule out organic causes for the decrease of vision in a preverbal child. With the ability of OCT to provide detailed chorioretinal structural assessment, it can act as an excellent tool for foveal assessment in the paediatric age group [18]. Macular lesions appearing to involve the fovea on fundus examination may have normal foveal depression on OCT [19]. Therefore, it is wise to do an OCT-aided foveal assessment along with a fundus examination in a paediatric patient with a history of ocular trauma. In a post-traumatic aphakic patient (Case 1) in our study, MIOCT revealed a macular scar involving the fovea with foveal atrophy and disorganisation of the outer retina ruling out amblyopia (secondary to aphakia) as the cause of low vision in this patient.

In our study, the mean TIAT for surgeons one and two was 211.75 ± 26.00 and 212.58 ± 23.47 seconds, respectively for all patients. Acquisition time varied according to retinal pathology for both surgeons. TIAT was the least for macular pathology and increased with cases that required peripheral/ anterior examination. In a pioneer study by Maldonado et al. the time needed to scan neonates, infants and children using HHOCT was less than 1 minute in 31% of sessions, between 1 and 2 minutes in 26% and 2 minutes in 43% of sessions [20]. While in a few imaging sessions in some children time to scan extended over 30 minutes in this pioneering study. In our study, the average TIAT was 3 minutes and 32 seconds, which was less than the average 7±5 minutes HHOCT in a study by Maldonado et al. [20]. The less average acquisition time in our study could be due to the OCT capture of retinal microstructure in real-time, a property of the MIOCT system. However, the absence of summed voxel projection (SVP) or live enface images, a property of HHOCT to provide a 2D image analogous to a fundus image adversely affected the TIAT in our study. As with the new generation, HHOCT image capture time has reduced to seconds, however, it still depends on patient cooperation and operator experience. Slightly more mean total image accusation time for the initial two cases for both surgeons could be attributed to the initial learning curve. The mean total image acquisition for both surgeons was comparable with no significant difference, suggestive of the good reliability of our novel technique.

MIOCT (RESCAN 700, Zeiss, Oberkochen, Germany) is a real-time intraoperative OCT system that is fully integrated into the microscope and controlled from the microscope foot pedal, allowing surgeons to take videos, snapshots and 3D OCT images without looking up or interrupting surgery. Snapshots and 3D OCT images are saved as DICOM files while videos can be saved in MPEG format. Real-time OCT allows choosing appropriate scan sizes as well as localising areas of interest. In our study, the 4x4 mm size scan size provided the best resolution and was best suited for macular pathology. 16x16 mm was the largest scan size possible, with the largest sweep area and was best suited for localising the area of interest. The DICOM files also allowed for quantitative analysis of change in the height of neurosensory detachment at the fovea pre- and immediate post-scleral buckling in a patient in our study (Case 6).

However, in our study, peripheral retinal pathology could not be imaged by this technique as “exo-illumination” failed to highlight the retina that was close to the equator of the eye. Despite manoeuvres to change the angulation of the external endoilluminator probe, the peripheral retina could not be imaged as the light emitted from the probe underwent scattering and diffraction from multiple interfaces before reaching the posterior segment. The inability to tilt the eyeball because of the requirement of coaxial placement of the MIOCT over a dilated pupil also made the examination of the retinal periphery extremely difficult. This limitation is not unique to our technique but is also seen with the commercially available HHOCT devices and all table-mounted non-widefield OCT devices that are used to image patients in the clinics [6]. The field of OCT acquisition using our technique can be increased by using 16 mm line scans for imaging relatively peripheral retinal pathology. Though this would decrease the resolution of the OCT image it could potentially enable the “exo-illumination technique” using MIOCT to provide good-quality OCT scans of the retina up to the equator. Another way out of this problem could be the future addition of SVP or live enface images to MIOCT allowing peripheral retinal imaging. The addition of live enface images to MIOCT may even obliviate the need for exo-illumination for retinal imaging.

This novel exo-illumination MIOCT technique allows retinal imaging in uncooperative paediatric patients planned for clinical EUA. In addition to this, exo-illumination MIOCT techniques can also be used in the post-surgical assessment of the retina in scleral buckling surgery. However, this technique requires general or local anaesthesia, which is not a requirement for either conventional table-mounted OCTs or HHOCTs. However, in clinics both conventional tablet-mounted OCT and HHOCT require patients to fixate for image acquisition which is nearly impossible in the uncooperative paediatric patient. Apart from uncooperative paediatric patients, this technique can also be used in uncooperative adult psychiatric patients or children with nystagmus where conventional OCT fails. Minimal acquisition time, negligible inter-operator variability, and good-quality image accusation can make the exo-illumination MIOCT technique a potent and reliable alternative to HHOCT. However further research is needed to this end. Our study has certain limitations. First, the sample size was small and did not cover the complete spectrum of retinal pathologies. Second, this study lacked a direct comparison of this novel technique with either conventional oct or HHOCT in paediatric patients.

## Conclusions

In conclusion, this novel study demonstrates a non-invasive technique for rapid acquisition of retinal OCT scans using MIOCT and “exo-illumination” in paediatric patients who are not cooperative for examination in the clinic. This technique provides real-time OCT images and circumvents the need for handheld OCT devices, thereby providing invaluable information during EUA and augmenting findings obtained from clinical examination of paediatric patients.
